# CD-CODE: crowdsourcing condensate database and encyclopedia

**DOI:** 10.1038/s41592-023-01831-0

**Published:** 2023-04-06

**Authors:** Nadia Rostam, Soumyadeep Ghosh, Chi Fung Willis Chow, Anna Hadarovich, Cedric Landerer, Rajat Ghosh, HongKee Moon, Lena Hersemann, Diana M. Mitrea, Isaac A. Klein, Anthony A. Hyman, Agnes Toth-Petroczy

**Affiliations:** 1 Max Planck Institute of Molecular Cell Biology and Genetics, Dresden, Germany; 2 Center for Systems Biology Dresden, Dresden, Germany; 3 Cluster of Excellence Physics of Life, TU Dresden, Dresden, Germany; 4 Dewpoint Therapeutics, Boston, MA USA

**Keywords:** Protein databases, Organelles

## Abstract

The discovery of biomolecular condensates transformed our understanding of intracellular compartmentalization of molecules. To integrate interdisciplinary scientific knowledge about the function and composition of biomolecular condensates, we developed the crowdsourcing condensate database and encyclopedia (cd-code.org). CD-CODE is a community-editable platform, which includes a database of biomolecular condensates based on the literature, an encyclopedia of relevant scientific terms and a crowdsourcing web application. Our platform will accelerate the discovery and validation of biomolecular condensates, and facilitate efforts to understand their role in disease and as therapeutic targets.

## Abstract (web summary)

Crowdsourcing condensate database and encyclopedia is a community-editable platform for verified biomolecular condensates and their protein constituents. It also provides an encyclopedia for the scientific terms used in condensate biology and a crowdsourcing web application.

## Main

Biomolecular condensates are membraneless organelles that selectively concentrate biomolecules (for example, proteins and nucleic acids) in the cell, with spatial and temporal precision^1^. In recent years, their role was implicated in several biochemical processes, in physiology and disease^2^. Consequently, biomolecular condensates are now leveraged as a new class of therapeutic targets^3,4^.

Basic science and drug discovery advances build upon published reports and the rate of new discoveries depends on timely accessibility to relevant data. However, as with every novel paradigm, new terms and concepts emerge and evolve as the field develops. Accordingly, currently available databases which catalog proteins involved in condensate formation use various definitions and criteria to define condensates and their constituent proteins and RNAs^5–8^. These are excellent databases curating proteins that phase separate. Specifically, LLPSDB^7^ and PhaSePro^6^ collect proteins that are thought to drive liquid–liquid phase separation, with the former curating exclusively in vitro data.

**Extended Data Fig. 1:**
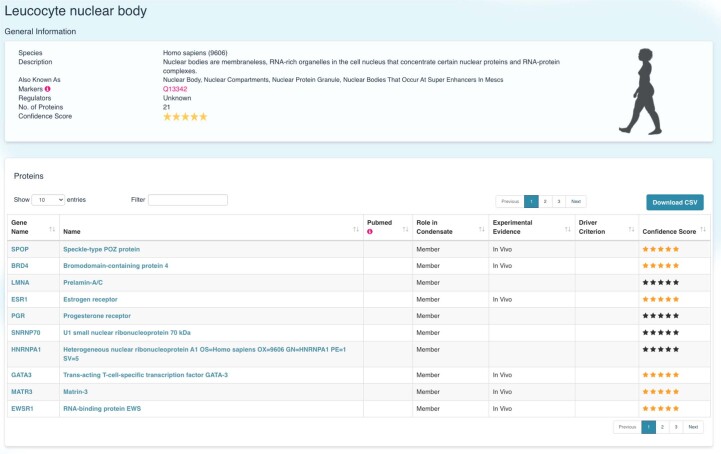
Detail page of the biomolecular condensate, Leucocyte nuclear body in humans. The top section displays ‘General Information’ of the condensate whereas the section below shows ‘condensate-specific protein’ data in a tabular format.

**Extended Data Fig. 2:**
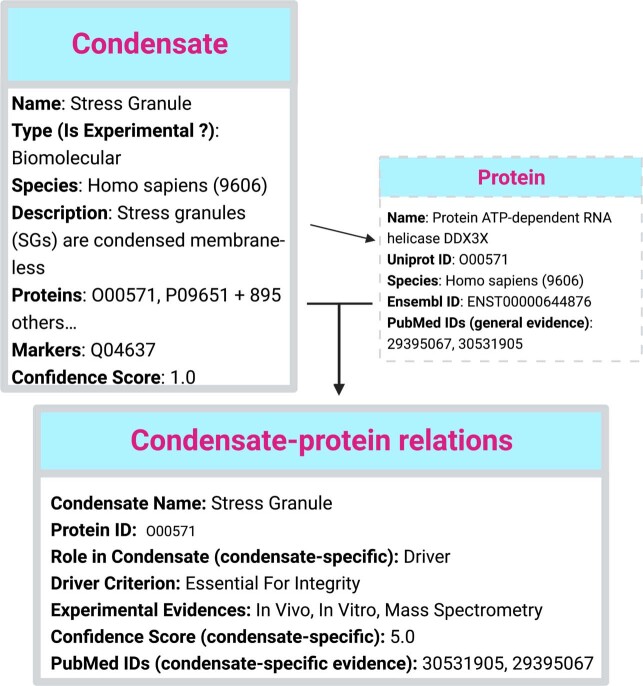
CD-CODE is a condensate centric database. Example data structure of a condensate and one of its member proteins. We defined two distinct entity collections – one for condensates and one for proteins, the latter comprising of general details regarding the members of the former. We also store mappings collection of condensate-protein relations, comprising of data points that relate to specific condensate-protein pairs.

**Extended Data Fig. 3:**
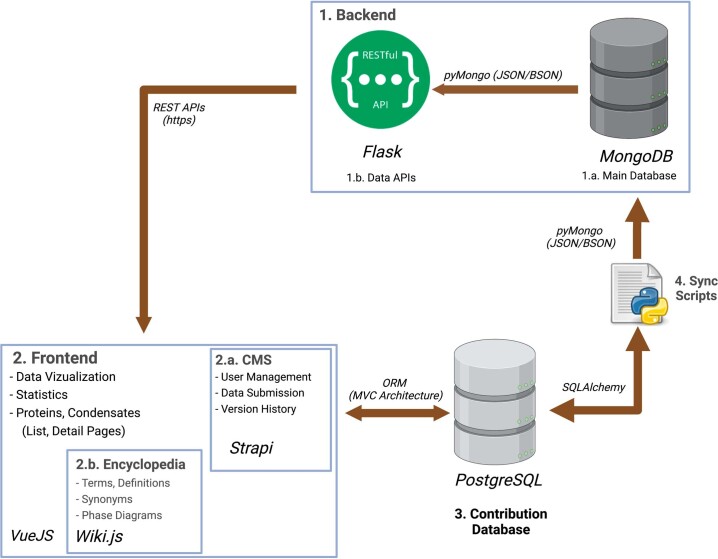
Schematics of the technical architecture of CD-CODE web application. The web application is internally divided into four distinct components. 1. The main database contains the condensates, proteins, and other related datasets. The API layer exposes consumable data for the frontend to visualize data. This can also be used for programmatic access to the data by statisticians and bioinformaticians. It also facilitates filter, search, sort, and some basic aggregate functionalities. 2. The frontend allows visualization of data, for example detail pages for condensates and proteins (Extended Data Figs. 1 and 7). A sub-component embedded within the frontend is the Content Management System (CMS) which facilitates user management, and workflow of each update item submitted by users. The CMS also helps maintain a history of contribution for each data record. 3. The contribution database stores the history of data from the crowdsourcing effort and helps versioning the database. All data edits submitted by contributors and review actions performed by maintainers are stored here to aid transparency. 4. The Sync script is a scheduled process that runs at regular time intervals (daily/weekly) to copy the update items safely to the main database. This python program interacts with both PostgreSQL from CMS and MongoDB from the main database in a producer-consumer paradigm.

However, these databases do not answer the following questions regarding biomolecular condensates: What are the biomolecular condensates discovered and verified to date? What are their known protein components? Which condensates is a given protein known to belong to? What are the experimental evidences supporting the existence of a particular condensate? Our goal is to generate answers for these and other important questions, and to create a community-editable database to facilitate the dynamic data updates. Therefore, we designed a condensate-centric database, which is based on the scientific literature, and provides experimental evidences, scores and references for each condensate–protein relationship (Extended Data Figs. 1 and 2). This database is updated dynamically by contributors to keep up with the growing knowledge in the field. We call our platform CD-CODE, which consists of three main parts: (1) a database of biomolecular condensates and their protein constituents; (2) an encyclopedia for the scientific terms used in condensate biology; and (3) a crowdsourcing web application (Extended Data Fig. 3).

**Fig. 1:**
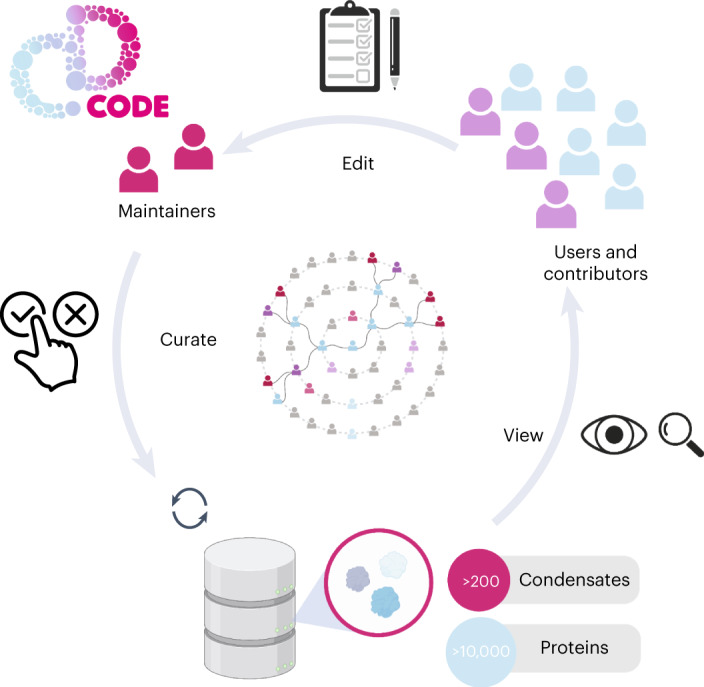
CD-CODE information flow. Users can view and search the data, or become contributors after registration and edit the content of the database via the community-editable web application. The maintainers assure quality control and only approved edits will be part of the dynamically updated database. Figure created with BioRender.com.

**Extended Data Fig. 4:**
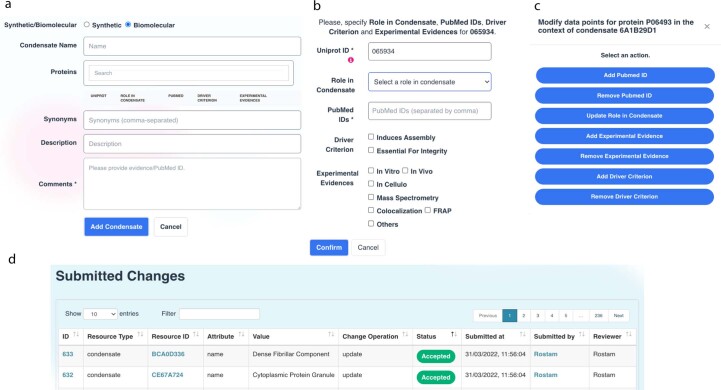
Data curation from the user interface. **a**) Add new condensate form. **b**) Add new protein form. **c**) Edit data actions. **d**) History of entries is recorded.

**Extended Data Fig. 5:**
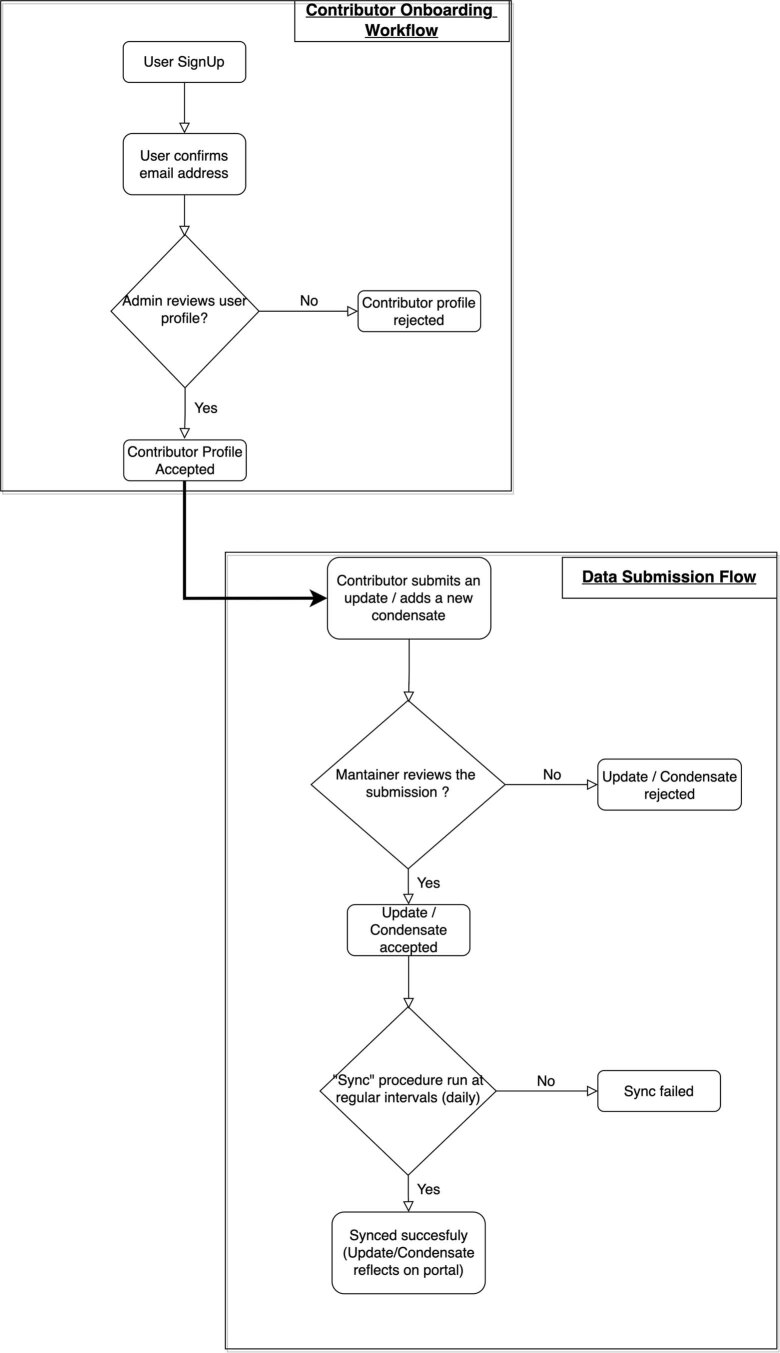
Flowchart of contributions. After submitting the registration form, the user receives an email to confirm the given address by clicking a link. After the user’s email address is confirmed, an administrator checks the pending contributor signup requests and approves them. A signed-in contributor can see editable attributes besides selected data points on detail pages, in addition to a new webpage form for submission of novel condensates. The new submissions are then moderated by maintainers first and later by the Sync script.

CD-CODE is a ‘living database’ designed for dynamic and rapid addition and review of information about condensates and proteins by users (Fig. 1) and is open to any expert researcher who wishes to contribute. Our user management system supports three types of users: viewers, contributors and maintainers. Viewers can read and download the curated information. Contributors can suggest edits and propose new condensate and protein entries (Extended Data Figs. 4 and 5). Maintainers are part of the development team, who curate the changes and accept or reject suggestions by contributors, who are then notified about the status of their suggestions and can engage in further discussion. To keep up with the rapidly evolving definitions, nomenclature and growing scientific evidence, the crowdsourcing platform allows the community to aggregate scientific findings in condensate biology.

**Extended Data Fig. 6:**
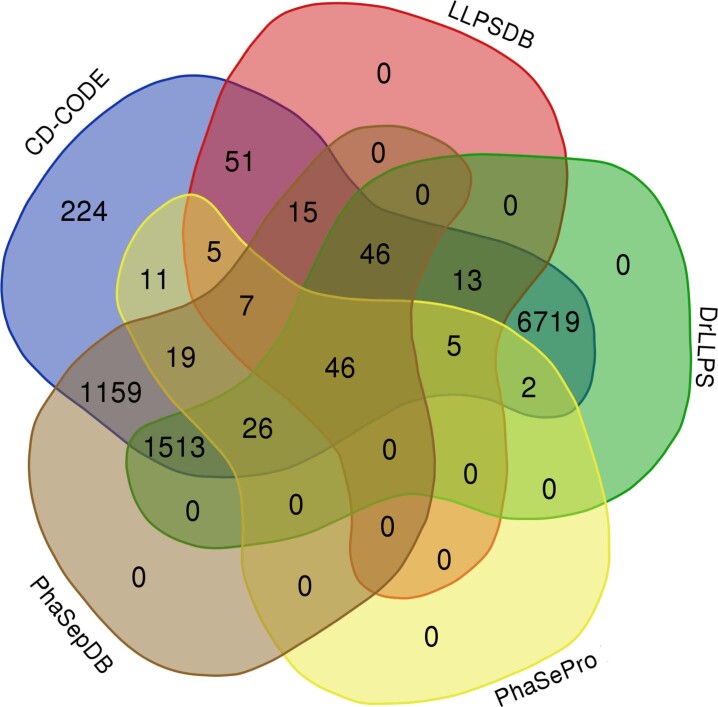
Venn diagram to compare protein entries in condensate/liquid-liquid phase separation (LLPS) databases. The Venn diagram displays the overlap of the protein entries in CD-CODE and four other LLPS/condensate databases (LLPSD^1^, DrLLPS^2^, PhaSepDB^3^, PhaSePro^4^). CD-CODE incorporates data from all four databases and additionally, there are 224 new condensate-forming proteins in v1.01 that were added to CD-CODE from curating the literature. Source data

**Extended Data Fig. 7:**
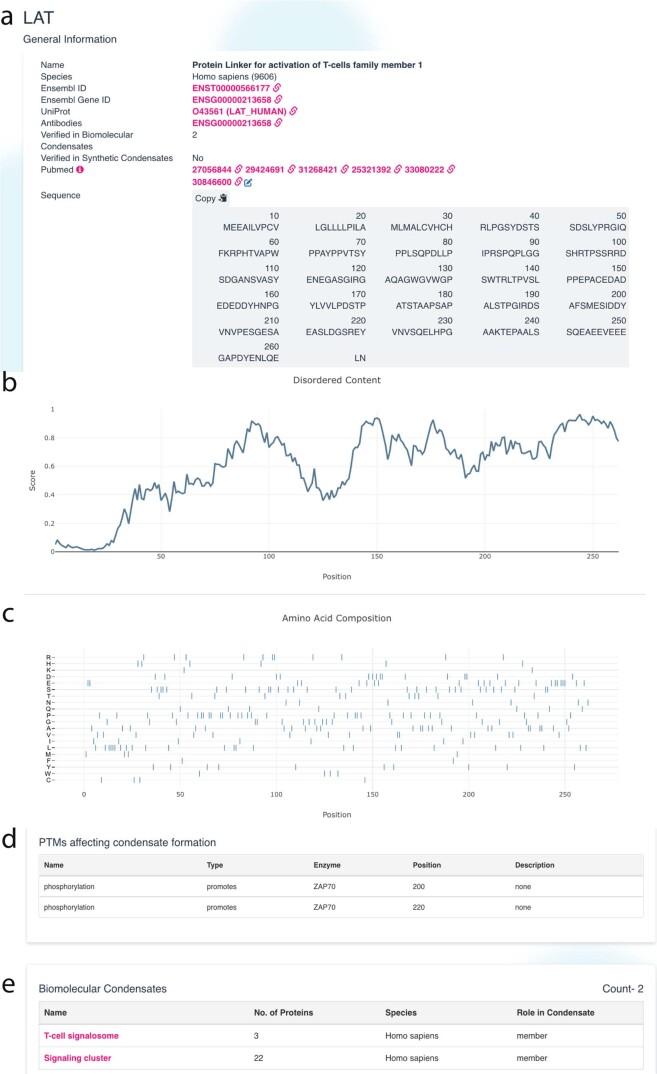
Example detail page to illustrate the general information and data points stored in CD-CODE. **a**) General information about a protein is shown for the LAT Linker for activation of T-cells family member 1 protein (Uniprot ID: LAT_HUMAN). Links are provided to other popular protein-related databases (Uniprot, Ensembl, Human Protein Atlas). **b**) Disorder score computed by IUPred^5^; **c**) Barcode plot depicting amino acid composition bias; **d**) Table of PTMs affecting condensate behavior; **e**) List of biomolecular condensates of which the given protein is a member.

At the time of this report, CD-CODE (cd-code.org) contains 9,861 proteins linked to 244 unique biomolecular condensates (and 375 in vitro synthetic condensates) across 49 different organisms. Notably, these numbers are continuously changing as contributors add and review more data. CD-CODE, as a semi-manually curated and annotated resource, aggregates information from the primary literature (to date, PubMed references published until 1 June 2022 were manually curated) and other databases^5–8^ (Extended Data Fig. 6 and Extended Data Tables 1 and 2). To promote easy integration with other resources, protein entries are cross-referenced with UniProt^9^, Ensembl^10^ and the Human Protein Atlas (proteinatlas.org)^11^. Common sequence properties of condensate proteins are also displayed graphically, such as disorder score^12^ and amino acid composition (Extended Data Fig. 7), facilitating the identification of regions that may drive condensate partitioning.

**Fig. 2:**
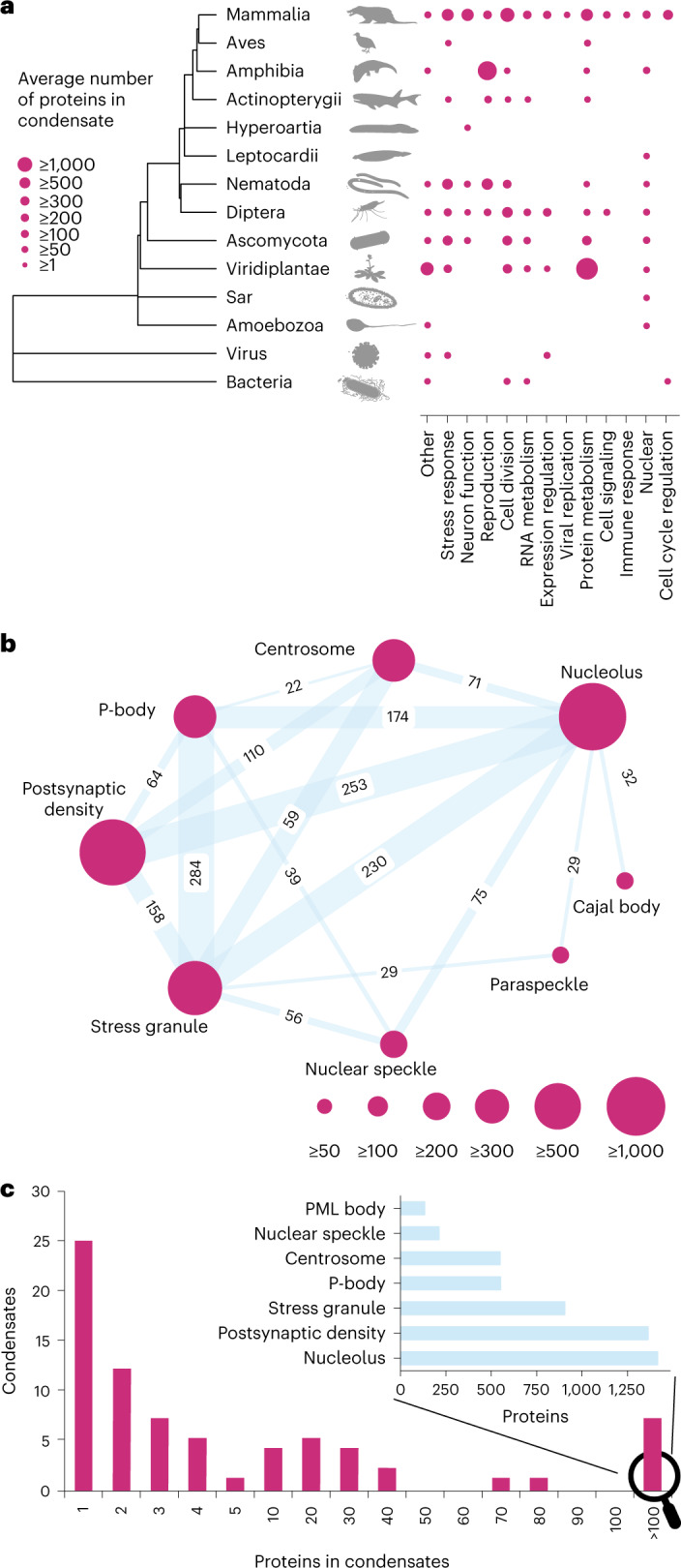
Selected features available at CD-CODE. **a**, Biomolecular condensates across the tree of life. CD-CODE contains information about 244 condensates across 49 species. Here, only major clades are shown for clarity and condensates were grouped into functional categories. **b**, Many proteins localize to multiple condensates. There is a large overlap between the proteomes of different biomolecular condensates in humans. The largest condensates in humans are represented as circles and the shared proteins between every two condensates are shown (only condensates with >20 connections are shown). **c**, The distribution of condensate proteome sizes in humans. Most biomolecular condensates have a few known protein members. The largest condensates contain >1,000 different proteins (inset). Source data

We standardized the names of condensates by creating an ontology from the literature and grouped condensates by functional categories (Supplementary Table 1) to reveal the evolutionary history of condensates. Most known condensates are found in mammals and many are clade-specific (Fig. 2a). Since our current knowledge is sparse and likely biased, the evolutionary origin of condensates remains an open future research direction that CD-CODE can facilitate.

While many proteins undergo liquid–liquid phase separation in vitro, it is unclear which proteins form condensates in cells and which condensates they partition into. To facilitate our understanding of condensate-specificity of proteins, we collected all known condensates a given protein was found in, and we curated the experimental evidence for association of each protein with a given condensate (confidence score, corresponding to zero to five stars: 1 star: literature evidence, PubMed identifier (ID); 2 stars, high-throughput; 3 stars, in vitro; 4 stars, in cellulo; and 5 stars, in vivo evidence). Condensates and proteins that have zero or one star rating have not been manually curated yet.

As expected, for dynamic cellular compartments, many proteins partition into different condensates and the overlap between condensate proteomes is substantial (Fig. 2b). While proteins may localize to multiple condensates (members), a few are obligate and essential components (drivers). We annotated 205 driver proteins in specific condensates, providing the corresponding experimental evidences. Our database revealed that several proteins that are drivers in one condensate are nonessential members of another (for example, G3BP1, a driver of stress granules, is also present in processing bodies (P-bodies) and neuronal ribonucleoprotein particle granules). CD-CODE will aid our understanding of the determinants of condensate-specific driver behavior, and whether a driver protein can be used as a ‘marker’ of a condensate in experiments.

Marker proteins are used to define the identity of the condensates and inform designing of condensate-targeting drug screening pipelines^3^. They are thought to be uniquely associated with a given condensate, and are commonly used to visualize condensates using microscopy, for example, in colocalization experiments to prove the localization of proteins into condensates. Our database revealed that several known marker proteins are not specific to a condensate. For example, whilst DCP1A is used as a marker for P-bodies, it also localizes to stress granules and nucleoli. Knowing specific protein components will facilitate the experimental design for accurate, specific identification of condensates.

CD-CODE enables us to answer the questions posed at the beginning: (1) there are currently 136 unique biomolecular condensates documented in the literature; (2) as an example, P-granules, which are the germ granules of *Caenorhabditis elegans*, have 190 documented protein components: one of them, pgl-3 (PGL3_CAEEL), is a driver for P-granule formation, and its presence within P-granules is supported by in vivo experimental evidence (5 star). Pgl-3 is exclusively reported to be associated with P-granules; thus, it is a P-granule-specific marker protein.

Databases that curate proteins undergoing liquid–liquid phase separation have facilitated the development of machine learning algorithms to predict phase separation^13–15^ and the discovery of what protein properties drive phase separation^15^. The next open question is which biomolecular condensate does a specific protein belong to. Our database contains a curated list of condensate proteomes (Fig. 2c), which can facilitate investigations of protein recruitment into specific condensates. Our resource can provide high-quality benchmarking data for machine learning algorithms aimed at predicting the protein components of condensates.

Furthermore, our comprehensive curation of condensate types and their respective composition in multiple species, and the level of experimental support, provides a valuable resource for drug hunters, which can inform the design of assays and screening pipelines. For example, in high-content imaging phenotypic screens, it is desired that the protein or protein combination chosen to be monitored is/are selective for the target condensate^3^. Additionally, through regular updating of the database by the community and via curation of new publications, CD-CODE supports and accelerates nomination of new condensate-associated drug targets.

The field of biomolecular condensates is highly transdisciplinary and ever-developing, where definitions and terms keep changing, creating a need for constant updates that require consensus within the community. The encyclopedia, as a standalone wiki, serves as a platform to aggregate knowledge about condensate research. In the future, we are planning weekly updates to integrate new data from the users, and yearly updates with new features and data points that become relevant to store, as the research field develops.

The main feature of CD-CODE is that it contains experimentally validated entries. However, caution should be exercised by users when interpreting lack of data on a particular protein, condensate or species, as this may simply reflect the biased interest of the community towards particular model systems and biological pathways. Any missing information could mean that (1) the protein or condensate has not been studied yet; (2) there is a research paper but the information has not been added to the database yet; (3) the condensate truly does not exist; or (4) the protein truly does not belong to a given condensate. As such, CD-CODE aims to highlight the unknowns in the field to guide future research questions to fill the gaps. These gaps in experimental evidence can be bridged by computational predictions^16,17^, which are beyond the scope of CD-CODE. Evolution of the CD-CODE database through ongoing curation of new experimental evidence will lead to a progressive increase in high-scoring condensate entries.

In summary, we present CD-CODE, a semi-manually curated condensate database, and a community-editable web application. The crowdsourcing platform allows the community to further scrutinize definitions and evidence as the field evolves. This will ensure that the ever-growing knowledge on condensate research is integrated into the database and into the encyclopedia in a timely manner.

## Methods

### Technical architecture of the web application

The web application is internally divided into four distinct components (Extended Data Fig. 3).

1. The main database contains the condensates, proteins and other related datasets. The database service used for this component is MongoDB, where the interlinking of resources is done at the logical layer. The Application Programming Interface (API) layer exposes consumable data for the frontend to visualize data. This can also be used for programmatic access to the data by statisticians and bioinformaticians. It also facilitates filter, search, sort and some basic aggregate functionalities. We used Flask, a simple lightweight Python framework, for this.
2. The frontend allows visualization of data—list and detail pages for condensates and proteins. It provides user-interactable controls for the addition/modification of data. The frontend is built using a Javascript framework called Vue.js. A subcomponent embedded within the frontend is the Content Management System (CMS), which facilitates user management and workflow of each update item submitted by users. The CMS also helps to maintain a history of contribution for each data record.
3. The contribution database stores the history of data from the crowdsourcing effort and helps versioning the database. All data edits submitted by contributors and review actions performed by maintainers are stored here to aid transparency.
4. The Sync script is a scheduled process that runs at regular time intervals (daily/weekly) to copy the update items safely to the main database. This python program interacts with both PostgreSQL from CMS and MongoDB from the main database in a producer–consumer paradigm.

### Data aggregation

The four most popular protein phase-separation databases are PhaSePro^6^, PhaSepDB^8^, LLPSDB^7^ and data resource of liquid–liquid phase separation (DrLLPS)^5^, which provide an excellent resource for phase-separating proteins along with their possible localization (membraneless organelles) and also synthetic condensates (in vitro experiments). However, all these databases are protein-centric and not condensate-centric, that is, a protein may or may not be part of a biomolecular condensate. To fill this gap and augment our current knowledge, we have built a condensate-centric database. At CD-CODE, we aggregated data from the four databases to create a seed dataset of condensates and their protein members (Extended Data Fig. 6). This was followed by a manual curation of the data and annotation of up-to-date evidences (PubMed references, experimental evidences, confidence scores explained below).

### Literature curation

All the 674 condensate literature-related PubMed references which were published after the release of the last four databases were checked manually, and new condensates and proteins involved in condensates were identified and added to CD-CODE along with the relevant PubMed IDs. In total, 26 new biomolecular condensates and 224 new proteins were added to CD-CODE which were not part of the previous databases (Extended Data Fig. 6 and Extended Data Tables 1 and 2).

### Data curation

Condensate names were standardized and condensates were merged based on synonyms that were manually assembled from the literature (Supplementary Table 1). In total, 9,916 protein–condensate relationships are supported by a PubMed ID as evidence.

### Annotation of driver proteins

We defined the ‘Role in condensate’ of a protein as the role it plays in a condensate formation and integrity. Based on their role in the formation of the condensate, proteins can be categorized into one of two classes—‘driver’ and ‘member’.

Drivers are defined based on the following criteria:

1. They induce the formation of a condensate.
2. They are essential for the integrity of a condensate.

Driver proteins are also called ‘scaffolds’ since they bind many other proteins. It is important to note that these annotations are valid in the context of a specific condensate. A protein acting as a driver for one condensate can be a member in another condensate.

### Confidence scores

We designed the confidence score criteria and started manually adding the experimental evidences. 1 star, PubMed reference-annotated; 2 stars, high-throughput experiment (for example, mass spectrometry); 3 stars, in vitro evidence; 4 stars, in cellulo evidence; 5 stars, in vivo evidence. We also collected the experimental methodology, for example, fluorescence recovery after photobleaching or microscopy colocalization.

### Crowdsourcing functionalities

Registered users can contribute to data curation, including any kind of attribute data update for any protein/condensate we already have at CD-CODE, such as marker addition, name change, description update, add/remove PubMed evidence and so on (Extended Data Fig. 4). Additionally, users can add new proteins to existing condensates and create new condensates by filling in a form.

### Encyclopedia

The encyclopedia (wiki.cd-code.org), another crowdsourcing element of CD-CODE, contains descriptive data related to definitions, synonyms and terminologies in the world of biomolecular condensates and liquid–liquid phase separation in biology. Contributors have the necessary credentials to create and edit content at the encyclopedia. It is powered by Wiki.js and provides easy content management functionalities to users who are not equipped with programming or HTML skills.

### Data analysis

Condensate counts for species were taken from CD-CODE v.1.01 and summarized by functional group annotation (Supplementary Table 1). The tree was downloaded from timetree.org (ref. ^18^) and scaled with the chronos function in the ape (5.6–2) R (v.4.2.0) package for better visualization. The tree was plotted using ggtree (v.3.4.4) and ggplot (v.3.4.0). Figure 2b,c was created using custom Python scripts (pandas: 1.2.4; matplotlib: 3.3.4; networkx: 2.5).

### Reporting summary

Further information on research design is available in the Nature Portfolio Reporting Summary linked to this article.

## Online content

Any methods, additional references, Nature Portfolio reporting summaries, source data, extended data, supplementary information, acknowledgements, peer review information; details of author contributions and competing interests; and statements of data and code availability are available at 10.1038/s41592-023-01831-0.

### Supplementary information

Supplementary Information Documentation.

Reporting Summary

Supplementary Table 1 Condensate nomenclature, synonyms and functional groups.

### Source data

Source Data Fig. 2 Source data for Fig. 2.

Source Data Extended Data Fig. 6 Source data for Extended Data Fig. 6.

## Figures and Tables

**Extended Data Table 1:**
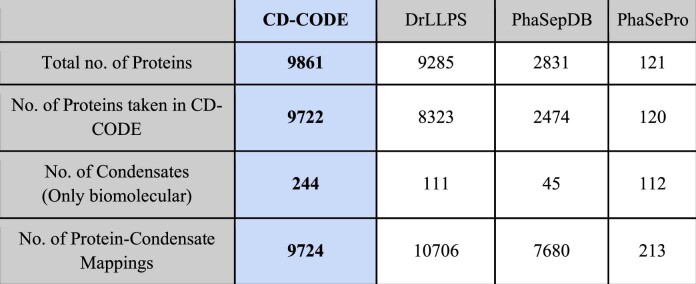
Biomolecular condensates across databases

Comparison of total number of data points presented by CD-CODE in comparison to other databases in the context of biomolecular condensates or membrane-less organelles (MLOs). We count the condensates from different species as separate counts of biomolecular condensates. The other three databases here also report phase-separating proteins without any condensate or with condensates named ‘Droplets’ and ‘Others’. Such entries are removed from CD-CODE by manual curation.

**Extended Data Table 2:**
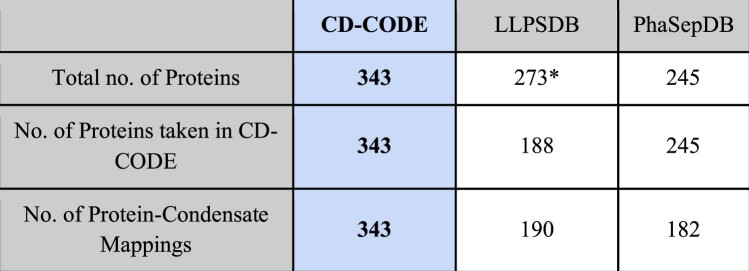
Synthetic condensates across databases

Comparison of total number of proteins presented in CD-CODE to other databases in the context of synthetic condensates or in *vitro* experiments. Since LLPSDB only reports *in vitro* experiments, the condensate-related counts are not reported for that.

*Out of the 273 proteins reported by LLPSDB, 198 are natural proteins, and 75 are designed proteins (not all experiments result in phase separation).

## Data Availability

Our database is available at cd-code.org and the encyclopedia at wiki.cd-code.org. Rolling feature update releases and data dumps will be noted here: https://cd-code.org/release. Source data are provided with this paper.

## Code availability

Software and documentation: https://git.mpi-cbg.de/dd-code-team/dd-code-docs.
