# Supplementary material for: CD-CODE: crowdsourcing condensate database and encyclopedia
Source: Nat Methods. 2023 Apr 6;20(5):673–6. doi: 10.1038/s41592-023-01831-0 (PMC10172118; doi:10.1038/s41592-023-01831-0)
Supplement: Supplementary file 1 — Documentation. [file 41592_2023_1831_MOESM1_ESM.pdf]

nature methods

Brief Communication

https://doi.org/10.1038/s41592-023-01831-0

CD-CODE: crowdsourcing condensate database and encyclopedia

In the format provided by the authors and unedited

Documentation

1. Technical Architecture

CD-CODE is a web application that presents a database of condensates and proteins with extended data update features in a community crowdsourcing style (**Fig. 1**). The distinct technical parts and sub-parts of CD-CODE are depicted in **Extended Data Fig. 3**. More detailed documentation can be found about the codebase of CD-CODE at https://git.mpi-cbg.de/dd-code-team/dd-code-docs.

1.1. Backend

At the heart of CD-CODE, sits the "main database" (numbered 1.a. in **Extended Data Fig. 3**) which contains the data of condensates and proteins. This database consists of verified data along with all the possible evidences including PubMed IDs. We used a community version of MongoDB 4.4 as the database server. The primary key of each collection is enforced using the "unique indexes" option. The attributes which are textual and informative in nature are indexed for tokenized text search which also powers the search and filter features from the frontend. The categorical attributes like species and condensate type are also indexes so as to facilitate faster-filtered queries.

A thin and lightweight layer of Application Programming Interface (API) (numbered 1.b. in **Extended Data Fig. 3**) sits on top of the main database to expose consumable data for both frontend applications and programmatic use. The APIs have been designed to be REST-compliant. The two main resources for the API are **"proteins"** and **"condensates"** respectively (**Extended Data Fig. 2**). For both of them, we have a list API – '[GET] /proteins' and '[GET] /condensates'. The list APIs have standard size-based pagination that accepts query parameters for page number and size of each page. They also allow filtering and sorting for a selected number of attributes. The tokenized search configured in the database also powers a text search filtering in the list APIs using the query parameter "query". Also, for both of these resources, there is a detail API – '[GET] /proteins/{uniprot_id}' and '[GET] /condensates/{unique_identifier}'. The list and the detail API power the respective list pages on the frontend as well.

We have used the Python framework Flask to develop our API layer and the package pyMongo to connect the API layer to the database. The APIs are also protected with an Authorization Bearer token which should be passed in the header of the request. The status codes of the responses are also REST-compliant.

1.2. Frontend

This component of CD-CODE contains all the GUI elements of the web application that a non-logged-in user would have access to. A simple user with just data view rights can see pages for proteins and condensates list and detail pages along with another page showing some statistics of the data. These user interfaces are built using the progressive Javascript framework Vuejs. This component directly consumes the REST APIs from the backend layer.

To facilitate the crowdsourcing functionality, we use an underlying layer of a headless content management system (CMS) powered by Strapi. Upon successful recruitment as a contributor (**Extended Data Fig. 5)**, the users get a modified user interface with edit options beside selected data points on the same UI (on detail pages). We term these contributions "UpdateItems". Using these interactive options, they can submit data modifications to CD-CODE. The contributors also get an additional form to create novel condensates and add their protein members along with the condensate-specific protein properties (**Extended Data Fig. 4**). The submissions from these forms are termed "NovelCondensates". We store these update items and novel condensates submissions in the database connected to the CMS by Strapi which is numbered 3 in **Extended Data Fig. 3.** For our purpose, we selected the open-source relational database PostgreSQL. The fixed data structure of the RDBMS influences the Strapi APIs and CRUD functionalities of the UpdateItems and NovelCondensates. At first impression, the CMS might appear to be like a redundant layer to support data write functionalities, however, it serves the following purposes:

- A layer of moderation for data edits
- Version control and history of data changes
- Record and reward the contribution by users

The encyclopedia (numbered 2.b. in **Extended Data Fig. 3**), another crowdsourcing element of CD-CODE, contains descriptive data related to definitions, synonyms, and terminologies in the world of biomolecular condensates and liquid-liquid phase separation in biology. The recruited contributors have the necessary credentials to create content in our encyclopedia. This is internally powered by Wiki.js and provides easy content management functionalities to users who are not equipped with programming or HTML skills.

1.3. Sync Scripts

The final component of CD-CODE is a scheduled process that runs in the background at regular intervals to copy the accepted data submission from the Contribution Database (PostgreSQL) to the Main Database (MongoDB). These are simple Python scripts that connect to each of the databases using an ORM connector and copy each update item one by one. The script performs quality checks regarding the feasibility of accommodating the updates, for example checking duplicity and data types. Additionally, a list of post-processing tasks run in the end to accommodate update related data points, for e.g. updating confidence scores based on evidence, or fetching data points from UniProt for entirely new proteins that did not exist in CD-CODE before.

2. Web Pages in CD-CODE

The following web pages are available in CD-CODE which display condensate-protein information:

1. Condensate List Page
    1. List of Biomolecular Condensates
    2. List of Synthetic Condensates
2. Condensate Detail Page
3. Protein List Page
4. Protein Detail Page
5. Statistics

Apart from these pages which display data from the actual main database, we also have the Encyclopedia which has descriptive information related to condensates and phase separation.

2.1. Protein Detail Page

The general information of each protein in CD-CODE is displayed via a protein detail page (**Extended Data Fig. 1**). For example, for the protein LAT_HUMAN, with UniProt ID "O43561", the protein detail page is present at the URL https://cd-code.org/protein/O43561. At CD-CODE, we identify proteins using the UniProt ID as the unique identifier (primary key), and thus, the URL for any protein detail page can be made by appending the respective UniProt ID at the end of https://cd-code.org/protein/{uniprot_id}.

2.2. Condensate Detail Page

The condensate page in CD-CODE shows information specific to a biomolecular or synthetic condensate along with the condensate-specific properties of the proteins present in it (**Extended Data Fig. 7**).

Each condensate entry is a record specific to a given species. Thus, for example, the Nucleolus of humans (9606) will have a separate entry compared to the Nucleolus of mice (10116). The species of each condensate is decided based on the protein members in it - if all the proteins belong to the same organism, that would be annotated as the species of the condensate. If there are multiple species for the proteins in the condensate, that is annotated as "Chimeras" (for synthetic condensates only). In addition, a list of manually curated marker proteins are shown for each condensate.

The protein table on a condensate detail page lists the known protein members of the condensate, along with a couple of general and specific protein properties. For easy identification of the protein. we also provide the gene and commonly known protein name along with the UniProt ID. The "functional_type" and "pubmed_ids" mentioned in this table are specific to the role of this protein in the given condensate. In addition to this, we also store "experimental_evidences" that serve as evidence for the existence of this protein in the condensate. There are seven possible values for such evidence at the moment - *"in_vivo",*

"in_vitro", "in_cellulo", "mass_spectrometry", "colocalization", "frap", and *"others"*. This column along with the "pubmed_ids" influence the confidence score of the protein being in this condensate. Another such attribute is known as "driver_criterion" is connected to the "functional_type" of the protein. If the protein is a driver, then "driver_criterion" states which of the three criteria were satisfied.

At the time of writing, we have 9916 protein-condensate mappings supported by one or more PubMed IDs as evidence. In addition to this, we have 226 experimental evidences, which were all obtained by manual curation.

2.3. Statistics

The real-time analysis of proteins and condensates in CD-CODE can be found on the "Statistics" page.

3. Crowdsourcing Functionalities

CD-CODE allows data write features in form of its crowdsourcing module. Users can contribute data using the enhanced interactive portal which can be accessed once a user has been onboarded as a contributor (**Extended Data Fig. 5**). The data submissions from contributors, after rounds of manual and automated moderation, are incorporated into the existing database. This unique module of CD-CODE allows the growing condensate community to come together and collaborate on knowledge (**Fig. 1**).

3.1. User Management

Besides, the usual viewer, CD-CODE allows users to signup and create profiles as registered members. There are three possible roles a signed-up user could be assigned:

- **Contributors:** users who can submit data updates or novel condensates. However, each submission is subject to moderation and it may or may not be integrated in the main database. Users can signup and submit requests to be given contribution rights. Post-verification of the profile, users are accepted as contributors. After signing in as a contributor, the UI of the protein and condensate details gets slightly enhanced with features to edit a given attribute. Also, new tables showing the history of data updates are visible to them.
- **Maintainer**: These are users who are responsible for accepting or rejecting the data submitted by contributors. Maintainer rights are available to a selected group of users. Usually, they are guided by a board of senior members from the condensate community. Maintainers have slightly different UI than contributors and controls to accept/reject contributions. Maintainers can submit data updates like contributors, which would be subject to another approval from another maintainer.
- **Administrator:** These users are not involved in data management but in user management. They do not have the right to submit or approve data updates. However, they can accept/reject profile signup requests and also deactivate a user if needed.

3.2. Contributor Onboarding and Workflow

A user willing to contribute to CD-CODE needs to be registered and recognized. We acknowledge the contributors on respective protein and condensate detail pages using their username.

Contributors can perform the following data update actions using the enhanced interactive controls on the detail pages. Each of the data updates is molded into a record called "UpdateItem" and stored in a relational database table. Each data update item is atomic in nature, i.e., it writes to a single attribute of a single data entity (protein or condensate).

The workflow for submission of entirely new condensates is also similar. However, the form accepting them asks for multiple data points - name, description, synonyms, and also proteins and related condensate properties for each protein. We store each condensate submission into a record called "NovelCondensates" and store them in another table.

The data structure of both UpdateItems and NovelCondensates has an attribute called "status" which influences the journey from submission to incorporation in the main database. Initially, a record is in "Submitted" status. After a maintainer's action, it can be changed to "Accepted" or "Rejected". After this stage, the sync scripts run on each data submission post and the status can be changed to "Synced" or "Failed". The sync process can change the status to "Failed" due to any errors that arise within the script.

Contributors can perform the following data update actions using the enhanced interactive controls on the detail pages. Each of the data updates is molded into a record called "UpdateItem" and stored in a relational database table. Each data update item is atomic in nature, i.e., it writes to a single attribute of a single data entity (protein or condensate).

1. Add/Remove "pubmed_ids" of a protein from Protein Detail page.
2. Add/Remove a "protein" from ProteinsTable of condensate from Condensate Detail page.
3. Add/Remove "marker" protein(s) of condensate from Condensate Detail page.
4. Add/Remove "regulator" protein(s) of condensate from Condensate Detail page.
5. Update "description" of condensate from Condensate Detail page.
6. Add/Remove "pubmed_ids" of a protein in condensate from Condensate Detail page.
7. Update functional_type of a protein in condensate from Condensate Detail page.
8. Add/Remove "experimental_evidences" of a protein in condensate from Condensate Detail page.
9. Add/Remove "driver_criterion" of a protein in a condensate from the Condensate Detail page.

References

1. Li, Q. *et al.* LLPSDB: a database of proteins undergoing liquid-liquid phase separation in vitro. *Nucleic Acids Res.* **48**, D320–D327 (2020).

2. Ning, W. *et al.* DrLLPS: a data resource of liquid-liquid phase separation in eukaryotes. *Nucleic Acids Res.* **48**, D288–D295 (2020).
3. You, K. *et al.* PhaSepDB: a database of liquid-liquid phase separation related proteins. *Nucleic Acids Res.* **48**, D354–D359 (2020).
4. Mészáros, B. *et al.* PhaSePro: The database of proteins driving liquid-liquid phase separation. *Nucleic Acids Res.* **48**, D360–D367 (2020).
5. Mészáros, B., Erdos, G. & Dosztányi, Z. IUPred2A: context-dependent prediction of protein disorder as a function of redox state and protein binding. *Nucleic Acids Res.* **46**, W329–W337 (2018).
